# Previous Cardiac Surgery: a Predictor of Mortality in Aortic Valve Replacement?

**DOI:** 10.21470/1678-9741-2018-0251

**Published:** 2019

**Authors:** Victor Dayan, Maria José Arocena, Amparo Fernandez, Eloísa Silva, Diego Pérez Zerpa

**Affiliations:** 1 Instituto Nacional de Cirugía Cardíaca. Montevideo, Uruguay.

**Keywords:** Previous Cardiac Surgery, Aortic Valve Replacement, Mortality, Survival

## Abstract

**Introduction:**

Previous cardiac surgery (PCS) is a risk factor for operative mortality in pa-tients undergoing reoperative aortic valve replacement (AVR) and may be influenced by the volume of patients in each center. The aim of this study was to evaluate the results of AVR in patients with previous cardiac surgery in a low volume cardiac center (400 cases per year).

**Methods:**

Between January 2006 and December 2016, 854 patients underwent isolated AVR surgery at our institution. Of these, 70 had PCS. Propensity match (PM) was per-formed to balance basal covariates. Operative mortality and survival were the primary outcomes.

**Results:**

The PCS and first-time surgery (FTS) groups had significant differences in base-line characteristics (PCS group were older, higher incidence of hypertension, endocarditis, NYHA III/IV, lower LVEF, higher creatinine and higher EuroSCORE). In the unmatched population, patients with PCS had higher operative mortality (17.1% *vs*. 4.6%, *P*=0.001). In the PM groups, this difference was not significant (12.5% and 3.6%, *P*=0.08). The only independent predictors for operative mortality found in the PCS group were age and fe-male gender. Age and diabetes were identified as the only independent predictors of sur-vival.

**Conclusion:**

PCS was not a predictor for operative mortality nor long-term survival in pa-tients undergoing isolated aortic valve replacement.

**Table:** 

Abbreviations, acronyms & symbols				
AF	= Atrial fibrillation		ICU	= Intensive Care Unit
AVR	= Aortic valve replacement		LVEF	= Left ventricular ejection fraction
AXC	= Aortic cross-clamp		NYHA	= New York Heart Association Functional Class
CABG	= Coronary artery bypass grafting		PCS	= Previous cardiac surgery
CPB	= Cardiopulmonary bypass		PM	= Pacemaker
FTS	= First time surgery		STS	= Society for Thoracic Surgeons
HC FMUSP	= Hospital das Clínicas da Faculdade de Medicina da Universidade de São Paulo		TAVR	= Transcatheter aortic valve replacement
HRs	= Hazard ratios		VIVID	= Valve in Valve International Data Registry

## INTRODUCTION

Previous cardiac surgery (PCS) has been considered a predictor for adverse outcomes in patients undergoing aortic valve replacement (AVR). This may be due to the higher risk profile of patients that are submitted to a second surgery compared to those with first time surgery (FTS) and that the technique for reoperation is generally more demanding^[1,2]^. Reoperation, either in patients with previous prosthetic valve replacement^[3]^ or coronary artery bypass grafting (CABG)^[4]^have not proven to be risk factors for operative mortality in re-do AVR^[5]^. Nonetheless, PCS is regarded as a risk factor for operative mortality in mortality scores such as EuroSCORE and STS.

Decisions during first time surgery such as type of prosthesis or treatment of minimally diseased valves at the time of CABG may be influenced by the potential risk of a subsequent cardiac surgery^[6]^. The potential risk of a reoperation for AVR is one of the main arguments for performing TAVR^[7]^.

Operative mortality has been shown to be very similar in patients undergoing first time and redo AVR^[4]^. Nonetheless, most of these data come from big centers with high volume of cases performed by surgeons with high level of expertise. Little data has been published regarding the risk of previous cardiac surgery in low volume centers which are the most frequent scenarios in South America.

The aim of this study was to evaluate the results of aortic valve replacement in patients with PCS in a low volume institution which performs 400 cases per year.

## METHODS

Patients who underwent isolated AVR from January 2006 until December 2016 were selected from our institution database. Urgent and emergency cases were excluded.

Included patients were divided according to PCS or FST. Basal and operative variables were extracted.

Propensity matching (PM) was used to balance the covariates. The following covariates were included in the propensity match: age, logistic EuroSCORE (European System for Cardiac Operative Risk Evaluation), body mass index, gender, pulmonary disease, neurologic disease, renal disease, hypertension, left main stem disease, diabetes, left ventricular ejection fraction (LVEF), New York Heart Association classification, previous myocardial infarction, endocarditis, creatinine. Propensity score for PCS was estimated by logistic regression. Variables included in the PM were chosen by clinical relevance. The treated observations were matched in a 1:1 ratio using the nearest-neighbor method with a caliper width of 0.1 of the standard deviation of the propensity score logit. Despite sequential modeling, EuroSCORE remained unbalanced so this variable was incorporated in the multivariable analysis.

Operative mortality was defined as death within 30 days of surgery or after 30 days during the same hospitalization subsequent to the operation. PCS was defined as any type of surgical procedure that required opening of the pericardium. PCS as a predictor of operative mortality was evaluated using logistic regression analysis in the propensity matched population. Regression analysis was also performed in the PCS group to identify predictors of operative mortality in this group of patients.

Kaplan-Meier survival curves were plotted for the matched and tested using a log-rank test. To analyze the association between risk factors and survival among the propensity-matched cohorts, each covariate was tested for prediction using a Cox proportional hazards. All covariates with a *P* ≤ 0.1 as well as those unbalanced were included in the multivariate model. Hazard ratios (HRs) were calculated for each variable.

## RESULTS

During the analyzed period, 854 (70 patients with PCS) patients underwent isolated AVR at our institution. Patients with PCS were older, displayed higher incidence of hypertension, endocarditis, NYHA III/IV, lower LVEF, higher creatinine and higher euroSCORE (Table 1). Patients with PCS had higher operative mortality (17.1% *vs*. 5.2%, *P*<0.001) and postoperative bleeding (761±851ml *vs*. 661±766ml, *P*=0.3) (Table 1).

**Table 1:** Clinical variables of the overall population (n=854).

	Previous surgery (70)	First surgery (784)	*P*
Age (years, SD)	62.3 (15.1)	67.1 (13.4)	0.012
Female (%)	27 (38.6)	368 (46.9)	0.179
Smoker (%)	17 (24.3)	176 (22.4)	0.741
Diabetes (%)	19 (27.1)	137 (17.5)	0.045
Hypertension (%)	43 (61.4)	541 (69.0)	0.191
Stroke (%)	3 (4.3)	23 (2.9)	0.347
Endocarditis (%)	10 (14.3)	34 (4.3)	<0.001
Atrial Fibrillation (%)	7 (10.0)	37 (4.7)	0.061
NYHA III/IV (%)	28 (40.0)	277 (35.3)	0.001
LVEF (%)	52.3 (14.6)	57.0 (10.6)	0.017
Creatinine (mg/dl)	1.28 (1.04)	1.03 (0.58)	0.03
AXC (min)	74.1 (24.6)	61.4 (18.7)	<0.001
CPB (min)	116.4 (46.2)	85.3 (29.6)	<0.001
Total bleeding (ml)	761 (851)	661 (766)	0.300
Postoperative stroke (%)	2 (2.9)	12 (1.5)	0.148
Postoperative AF (%)	14 (20.0)	242 (30.9)	0.057
Permanent PM (%)	3 (4.3)	46 (5.9)	0.666
ICU stay (days, SD)	3.1 (3.2)	2.6 (3.9)	0.395
euroSCORE (SD)	16.4 (13.4)	6.7 (6.2)	<0.001
Operative mortality (%)	12 (17.1)	41 (5.2)	<0.001

AF=atrial fibrillation; AXC=aortic cross-clamp; CABG=coronary artery bypass grafting; CPB=cardiopulmonary bypass; ICU=Intensive Care Unit; LVEF=left ventricular ejection fraction; NYHA=New York Heart Association Functional Class; PM=pacemaker

PM rendered 112 patients with similar baseline characteristics except for higher incidence of endocarditis, atrial fibrillation and EuroSCORE in the PCS group which were adjusted by logistic regression analysis (Table 2). In the matched population, operative mortality was similar in PCS and FST surgery groups (12.5% *vs*. 3.6%, *P*=0.08) (Table 2). AXC, CPB time and ICU stay were significantly higher in the PCS group.

**Table 2:** Clinical variables of the propensity matched population (n=112).

	Re-do (56)	First surgery (56)	*P*
Age (years, SD)	63.1 (14.5)	67.7 (14.9)	0.098
Female (%)	22 (39.3)	25 (44.6)	0.566
Smoker (%)	14 (25.0)	8 (14.3)	0.154
Diabetes (%)	12 (21.4)	7 (12.5)	0.208
Hypertension (%)	33 (58.9)	38 (67.9)	0.327
Stroke (%)	2 (3.6)	2 (3.6)	1
Endocarditis (%)	5 (8.9)	0 (0)	0.022
Atrial Fibrillation (%)	6 (10.7)	0 (0)	0.012
NYHA III/IV (%)	18 (32.2)	23 (41.1)	0.534
LVEF (%)	52.5 (14.5)	53.5 (13.3)	0.719
Creatinine (mg/dl)	1.15 (0.53)	1.02 (0.35)	0.107
Previous CABG (%)	18 (32.1)	-	-
Previous valve surgery (%)	25 (44.6)	-	-
Previous aortic surgery (%)	4 (7.1)	-	-
Previous congenital surgery (%)	8 (14.3)	-	-
Other previous surgery (%)	1 (1.8)	-	-
AXC (min)	72.6 (23.1)	42.5 (10.3)	<0.001
CPB (min)	112.1 (44.2)	68.8 (19.4)	<0.001
Bioprosthesis	35 (62.5)	46 (82.1)	0.09
Total bleeding (ml)	867 (898)	649 (620)	0.140
Postoperative stroke (%)	1 (1.8)	2 (3.6)	0.558
Postoperative AF (%)	14 (25.0)	16 (28.6)	0.670
Permanent PM (%)	2 (3.6)	1 (1.8)	0.558
ICU stay (days, SD)	3.3 (3.2)	2.3 (1.6)	0.036
EuroSCORE (SD)	15.1 (12.1)	6.4 (4.9)	<0.001
Operative mortality (%)	7 (12.5)	2 (3.6)	0.082

AF=atrial fibrillation; AXC=aortic cross-clamp; CABG=coronary artery bypass grafting; CPB=cardiopulmonary bypass; ICU=intensive care unit; LVEF=left ventricular ejection fraction; NYHA=New York Heart Association Functional Class; PM=pacemaker

After adjusting for baseline differences in the PM population, PCS was not an independent predictor for operative mortality.

Predictors for operative mortality were evaluated in patients with PCS (Table 3). The only independent predictors in this group of patients were age and female gender. Type of previous surgery (CABG or valve replacement) was not a predictor for operative mortality.

**Table 3:** Independent predictors for operative mortality in previous cardiac surgery patients undergoing aortic valve replacement (n=70).

Variables	OR (95%CI)	*P*
Age	1.15 (1.03-1.28)	0.016
Female	8.5 (1.1-66.7)	0.041

**Fig. 1:**
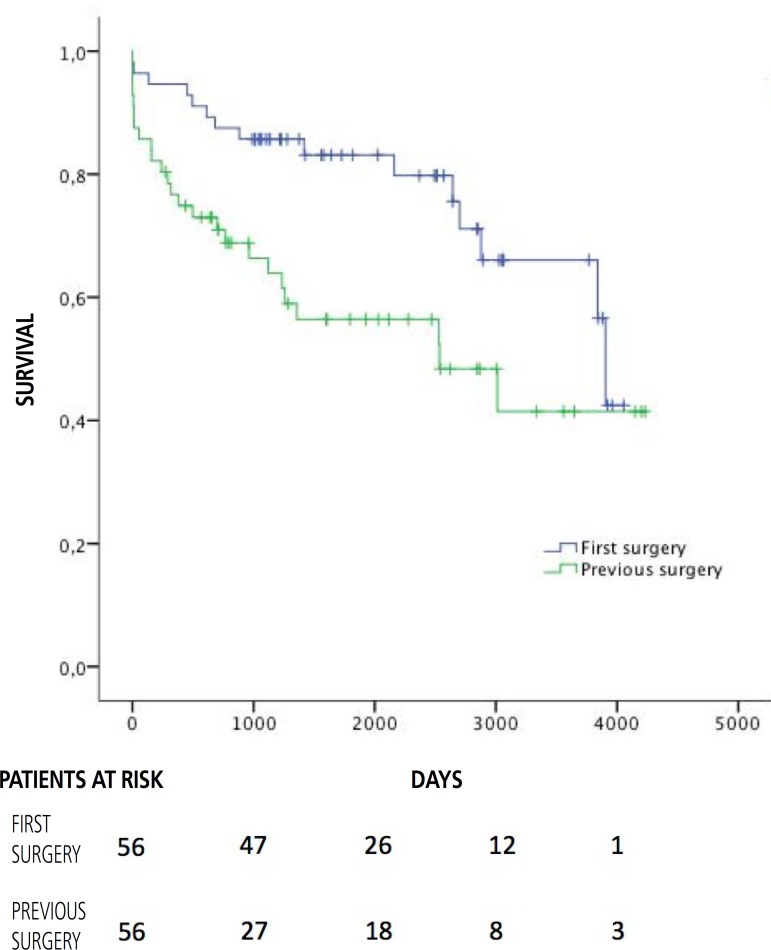
Survival in the matched population.

Among the PM population, survival was significantly lower in the PCS group (6.52 ± 0.72 years *vs*. 8.72 ± 0.3 years, *P*=0.013) (Figure 1). Nonetheless, after multivariate Cox regression analysis, age and diabetes were the only independent predictors of survival (Table 4).

**Table 4:** Independent predictors for survival in propensity matched patients with isolated aortic valve replacement (n=140).

Variables	HR (95%CI)	*P*
Age	1.07 (1.01-1.12)	0.012
Diabetes	2.78 (1.03-7.51)	0.045

## DISCUSSION

This study demonstrates that PCS is not a predictor for operative mortality nor long-term survival in propensity matched patients undergoing isolated AVR. The only independent predictors for survival in PM patients undergoing isolated AVR were age and diabetes.

Analysis of the overall population showed that patients with PCS were younger but with more risk factors such as diabetes, worse LVEF, higher incidence of endocarditis, worse NYHA class and higher preoperative creatininemia. The higher risk profile of these patients is reflected by their higher EuroSCORE compared with patients undergoing first time AVR.

It is well known that reoperation is a highly demanding procedure performed in sicker patients which requires high surgical expertise^[2]^. Therefore, we believe that smaller cardiac surgery centers should rely on results provided by similar centers instead of underestimating the operative mortality of these procedures based on published results from high volume centers. Results from big surgical centers such as the Mayo Clinic, report low operative mortality in previous AVR patients and therefore conclude that bioprosthesis should be encouraged in younger patients^[3]^. In their study, the authors do not specify the surgical risk score (EuroSCORE or STS score) of their population nor the inclusion of patients who had previous CABG in the reoperative AVR group. These data are very important in order to define the real surgical risk of the reoperative group. Even though our center has a limited number of redo cases per year, operative mortality in the entire cohort was similar to larger regional cardiac centers^[8]^. Pomerantzeff et al.^[8]^ at InCor HC FMUSP report 18% operative mortality in their entire redo aortic valve replacement group which is quite similar to our data. In the PM cohort, the re-do group had a higher risk mean EuroSCORE with a predicted operative mortality of 12%. The observed operative mortality of our previous surgery and first-time propensity matched groups was similar to the predicted EuroSCORE. Therefore, results from our center reflect appropriate standard of care and have external validity implications.

We analyzed specifically the predictors for operative mortality in patients with PCS. The only independent predictors after multivariate analysis were age and gender. Jamieson et al.^[9]^ published similar findings regarding the risk of female gender in this group of patients. Although we were not able to demonstrate it due to our low number of patients, we believe that the use of smaller prosthesis and hence higher risk of severe patient-prosthesis mismatch in female patients could explain the higher operative mortality. Lytle et al have shown similar operative mortality results as our group and have established as well, age and female gender as independent predictors of mortality^[10]^.

Reoperative aortic valve replacement requires to be performed by trained surgeons. Difficulty in these procedures lie on the risk of damaging anatomical structures during chest re-entering or dissection of pericardial adhesions. Furthermore, these patients have higher risk of permanent pacemaker due to complications associated with prosthesis explantation^[3]^. Nonetheless, our results show very low incidence of pacemaker requirement, which was similar to the primary AVR group.

Long-term survival is lower in patients with PCS probably due to their higher risk profile and not to the re-operation procedure. After adjusting for these comorbidities, predictors for long-term survival were similar to major cardiovascular risk factors: age and diabetes. Therefore, patients who survive the surgical procedure depend on the adequate control of their risk factors to ensure long-term survival.

This data should encourage careful decision-making as to whether choosing to operate on patients with a PCS, and considering other less invasive therapeutic options if indicated, such as TAVR^[11]^. The VIVID (Valve in Valve International Data Registry) registry has reported 1-year survival of 83.2% in patients who received a TAVR for aortic bioprosthesis degeneration^[12]^which is similar to our 1 year survival in the PCS group (80.3%). Predictors for higher 1-year mortality in the VIVID registry were small aortic bioprosthesis (≤ 21mm) and aortic stenosis.

This study suffers from a number of limitations, including the heterogeneity of the study group, the relatively small sample size (total sample size 854 patients, with 70 patients in the reoperation group) and the limitations inherent to the retrospective nature of the analysis. Although PM was performed to correct for these differences, selection bias is inherently introduced due to the sample characteristics. The volume of cardiac surgeries performed in our institution, which is around 400 per year, may also influence the results.

## CONCLUSION

Higher comorbidities in patients with PCS explain the higher risk for operative mortality and long-term survival in reoperation for AVR. Nonetheless, in PM patients, PCS is not predictor for operative mortality nor long-term survival. Our results show that re-do AVR may be safely performed in a low volume cardiac surgery center.

**Table:** 

Authors' roles & responsibilities	
VD	Substantial contributions to the conception or design of the work; or the acquisition, analysis, or interpretation of data for the work; drafting the work or revising it critically for important intellectual content; final approval of the version to be published
MJA	Substantial contributions to the conception or design of the work; or the acquisition, analysis, or interpretation of data for the work; drafting the work or revising it critically for important intellectual content; final approval of the version to be published
AF	Substantial contributions to the conception or design of the work; or the acquisition, analysis, or interpretation of data for the work; drafting the work or revising it critically for important in-tellectual content; final approval of the version to be published
ES	Substantial contributions to the conception or design of the work; or the acquisition, analysis, or interpretation of data for the work; drafting the work or revising it critically for im-portant intellectual content; final approval of the version to be published
DPZ	Substantial contributions to the conception or design of the work; or the acquisition, analysis, or interpretation of data for the work; drafting the work or revising it critically for important in-tellectual content; final approval of the version to be published
